# Substance use determinants in Jamaican under-25s: family, peers, spirituality and maltreatment (literature review)

**DOI:** 10.1192/bjo.2021.762

**Published:** 2021-06-18

**Authors:** Oliver Sargent, Mayeh Omar

**Affiliations:** 1 Medical Student, University of Leeds; 2 Nuffield Centre for International Health and Development, University of Leeds

## Abstract

**Aims:**

Jamaica is undergoing rapid change in its attitudes and laws regarding substance use; understanding the reasons why under-25s use substances will help inform future interventions and policy decisions. This review will investigate the determinants of substance use in under-25s in Jamaica, aiming to identify key sub-groups to target with interventions, and propose topics for further research.

**Method:**

A literature search was performed with Ovid on three databases, using wildcards and synonyms to increase the number of hits. This search produced 379 results, of which 41 remained after inclusion/exclusion criteria were applied. Additional sources were utilised as the review was written.

**Result:**

Strong family relationships are protective against illicit substance use for under-25s, with conflicting results for licit substance use. Healthy peer relationships protect against substance use, particularly in the academically-stressful university environment. All Jamaican under-25s appear to be susceptible to peer pressure, which increases the likelihood of substance use. Spirituality is protective against substance use, although male Rastafarians are more likely to use cannabis. Certain forms of childhood maltreatment make use of particular substances more likely. University students and under-18s brought up in single-parent families are key sub-groups to target with interventions. Further research on mechanisms by which these determinants work, particular religions and which determinant has the greatest effect is recommended.

**Conclusion:**

Various factors can protect against or predispose substance use in Jamaican under-25s. This review, and future research, can help inform policy decisions and intervention design for the key sub-groups found.

